# My Bad, You Got This: witnessing, therapist attitude and the synergy between psychedelics and inner healing intelligence in the treatment of trauma

**DOI:** 10.3389/fpsyg.2025.1469559

**Published:** 2025-01-15

**Authors:** Lawrence Fischman

**Affiliations:** Department of Psychiatry, Tufts University School of Medicine, Maine Medical Center, Maine Track Program, Boston, MA, United States

**Keywords:** inner healing intelligence, trauma, MDMA-assisted therapy, psychedelics, relational psychoanalysis, witnessing, dissociation, therapist attitude

## Abstract

The MAPS (Multidisciplinary Association for Psychedelic Studies) sponsored MDMA-assisted therapy protocol has had greater success in treating trauma in preliminary clinical trials than any prior psychotherapeutic, pharmacologic, or combined approach. It is predicated on a synergy between drug action and the participant’s inner healing intelligence. The latter is described mainly by analogy with the body’s capacity to heal itself, and the treatment is characterized as a means of activating or accessing this capacity. How is this rather mysterious-sounding process so effective? I suggest that the therapist’s full commitment to, and trust in this treatment framework, along with the medication’s subjective enhancement of trust, encourages individuals who have suffered trauma and have difficulty trusting others to engage the therapist as a kind of witness. I discuss parallels between the therapeutic attitude implied in the inner healing intelligence model and the way a therapist can act as witness in the resolution of dissociative enactment in relational psychoanalysis. Trusting the healing capacity of one’s inner healing intelligence is dynamically equivalent to trusting the relational process. This makes trusting one’s inner healing intelligence a process of feeling witnessed. In both settings, the therapist’s willingness to acknowledge her technical limitations or failings, coupled with a conviction that the participant/patient’s primary need in processing trauma is to feel witnessed, facilitates the integration of dissociated experience.

## Introduction

The first part of the 21st century has witnessed a revolution in the psychoanalytic understanding and treatment of trauma. In parallel, renewed research with psychedelics has witnessed the emergence of a powerful new protocol for the psychedelic-assisted psychotherapy of trauma. Both approaches rest on a theoretical model for altering patterns of dissociation. Notwithstanding the distinctive, arcane jargon in which these models are clothed, each approach emphasizes the importance of the attitude of the therapist/analyst in the act of witnessing as critical to its mutative potential.

The lead investigator in the MAPS (Multidisciplinary Association for Psychedelic Studies) sponsored MDMA-assisted treatment of Post-Traumatic Stress Disorder (PTSD) clinical trials, Michael Mithoefer, suggests that the medication and psychotherapy work synergistically. “The basic premise of this treatment approach is that the therapeutic effect is not due simply to the physiological effects of the medicine; rather, it is the result of an interaction between the effects of the medicine, the therapeutic setting and the mindsets of the participant and the therapists” (Mithoefer, 2017, p. 6). This may explain its dramatically higher treatment success rates compared with previous combinations of pharmacotherapy and psychotherapy, and with either procedure alone (Mitchell et al., 2021, 2023; Feduccia et al., 2019). Treatment success, here, refers to objectively measured reductions in PTSD symptomatology, but just as importantly, to subjectively assessed reductions in defensiveness, shame, guilt, and anger, as well as increased acceptance, flexibility, openness, and pro-sociality (Bedi et al., 2009; Dolder et al., 2016; Grinspoon and Doblin, 2001; van der Kolk et al., 2023; Wagner et al., 2017). Time will tell whether these impressive results^1^ will stand up in general use to the challenges of larger, more diverse treatment groups, more complex symptomatology and co-morbidity, inevitable and necessary cost-cutting strategies, and less well-trained and supervised practitioners, but with such unprecedented early success rates, we may well ask, how does this synergy work (Nayak and Johnson, 2021)?

The MAPS treatment model is based on a notion that MDMA enables individuals with PTSD to make use of one’s “inner healing intelligence,” a term that originated with Stan Grof (Grof and Grof, 2010) and has been developed further by Mithoefer (2022). O'Donnell et al. (2024) write,^2^ “The inner healing intelligence is a *capacity* and a *process*; crucially, it is not a material entity;” it refers to a person’s “innate tendency toward self-directed healing and growth.” A preliminary study to operationally define a related concept, the “inner healer,” lends some support to its validity as a construct, but the study is limited by being based on a single item (Peill et al., 2024). It is closely related to the philosophical concept of “entelechy,” the vital force directing one’s growth or life, or the realization of one’s potential, which Richards (2017) has applied to psychedelic-assisted psychotherapy, but this term is equally mysterious. In the following pages, I will outline parallels between the therapeutic attitude implied in the concept of inner healing intelligence and aspects of the therapeutic attitude regarded by psychoanalysts as crucial for enabling change in patients with histories of trauma. In both settings, the therapist’s attitude embodies many of the modifications to psychoanalytic technique pioneered by Ferenczi (1931, 1933/1988). I will discuss how this attitude works synergistically with psychedelics to loosen the trauma-induced discontinuities in consciousness commonly referred to as *dissociation*. I will discuss how both the drug and the listening attitude prescribed by these two psychotherapeutic techniques create an ambience suitable for the experience of *feeling witnessed*. Recognizing this common ground may contribute to a greater understanding of the treatment mechanisms involved and facilitate dialog between practitioners and theoreticians in both camps (Barnett et al., 2018, 2024; Davis et al., 2022; Kraiem et al., 2024).

## Inner healing intelligence and psychedelic-assisted psychotherapy

Psychedelic-assisted psychotherapies come in many forms. Roughly speaking, they may be divided into non-specific, non-directive, supportive therapies (Carhart-Harris et al., 2016, 2018; Davis et al., 2021; Griffiths et al., 2016; Mithoefer, 2017) and “evidence-based” treatments which are tied to specific psychotherapeutic modalities and often adapted for specific conditions (Bogenschutz and Forcehimes, 2017; Brennan and Belser, 2022; Carhart-Harris et al., 2021; Sloshower et al., 2020). While the MAPS PTSD protocol is generally described in the literature as “supportive” and characterizes itself as eclectic rather than emphasizing any particular psychotherapeutic tradition, this belies its nuanced, psychodynamically-informed management of dissociation, resistance, transference, countertransference, character, interpretation, and relational considerations. It rests on empowering a somewhat conjectural and un-psychoanalytic sounding psychic agency—the “inner healing intelligence”—but is this notion any less evidence-based than the psychoanalytic notion of an “ego”?

The MAPS treatment manual holds certain aspects of therapist conduct as indispensable to the healing process. These include:

1. The development of therapeutic alliance and trust over the course of therapy is essential.
2. A nondirective approach to therapy based on empathetic rapport and empathetic presence should be used to support the participant’s own unfolding experience and the body’s own healing process. A non-directive approach emphasizes invitation rather than direction.
3. It is essential to encourage the participant to trust their inner healing intelligence, which is a person’s innate capacity to heal the wounds of trauma. It is important to highlight the fact that the participant is the source of their own healing (Mithoefer, 2017).

The core principle of MAPS MDMA assisted therapy is often explained to study participants through an analogy with wound healing. “The doctor or nurse can help by removing obstacles, such as gravel and debris, and can create favorable conditions but it is always the person’s own healing intelligence that does the healing” (Mithoefer et al., 2014). This emphasizes that the participant has the capacity to heal herself, and that the therapists and medication are merely adjuncts to the participant’s inner healing intelligence. Accordingly, the manual is filled with examples of verbal exchanges between participant and therapist which illustrate how meaningful affective change and insight derive not from the therapists’ interpretations, but from providing an atmosphere of safety and encouragement which allows the participant’s healing process to unfold.

This approach is also captured in another analogy. “A seed has within it the intelligence to grow into a vibrant and blossoming plant” (Clare, 2018). In the same way that a nourishing environment (soil, water, light, and air) allows a seed/plant to flourish, providing a background of safety enhanced by MDMA in a therapeutic setting enables the participant “to connect with their innate ability to heal and grow, through developing a relationship with their inner healing intelligence and, from there, working through trauma” (Clare, 2018).

The clear message in these analogies is that the resources for healing lie within, not with the therapists nor with the drug; hence, the colloquial title of this paper, “You Got This.” The concept of inner healing intelligence implicitly recognizes that individuals who have experienced trauma are more likely to place trust in their own resources than in someone else’s. In one way, the MAPS manual seems geared to persuade therapists from psychodynamic and other training backgrounds that less is more, so to speak; that positive treatment outcomes hinge less upon the therapist’s knowledge and properly timed interpretation of the participant’s psychodynamic conflicts and transferences than acting as witnesses to the participant’s self-discovery. “In this approach, it is assumed that the therapists have no way of knowing when, or for whom, it will be most useful to focus on the “index trauma” and when it may be most useful for them to make connections to other events or periods in their lives. The relatively non-directive approach leaves this decision up to the individual’s innate healing intelligence” (Mithoefer, 2017).

A corollary of the inner healing intelligence model suggested by the above quote is that the healing process is regarded as a spontaneously unfolding inner experience. The therapist’s role, while critical to the emergence of this process, is purposefully minimized. This is evident in the way the therapist refers to the process, which helps to structure the process this way in the participant’s mind.

Participant: “I feel so crooked. Are you going to be able to walk me through any of the traumatic experiences to kind of help me focus?”

Therapist: “Absolutely. If it feels like it’s the time to do that now, we can help you do that, but it might be better at this point to first go inside and, as much as you can, relax into the way the experience will unfold. Sometimes talking can get in the way of the experience. We can talk more later” (Mithoefer, 2017, p. 41).

The therapeutic process is conceptualized as emerging through a rhythm determined by the participant’s inner healing intelligence. “The pace of the session allowing for the participant’s own process to unfold spontaneously is essential” (Mithoefer, 2017, p. 10). The therapists’ role in this respect is to gauge whether the participant is allowing herself to stay in tune with the naturally unfolding rhythm of the process, or perhaps anxiously intellectualizing. “This situation is sometimes referred to as the participant “getting ahead of the internal emotional experience.” In this situation, it is valuable for the therapists to intervene and guide the participant back to their internal experience” (Mithoefer, 2017, 40). The following is an example of such an intervention:

Therapist: “Maybe this would be a good time to put the eye shades and headphones on and go back inside **for a period of time**, to see what the medicine and your inner healing intelligence may show you about all of this (Mithoefer, 2017, p. 41; bold in original).

The art of psychotherapy in the MAPS manual mainly consists in this type of balancing: “To maintain the delicate balance between focusing on the inner experience and providing a safe space for exploring this experience in an open-ended way, the therapists must respect the inner healing intelligence of the participant’s own psyche and body, skillfully interweaving periods of interaction with periods of silent witnessing” (Mithoefer, 2017, p. 31). In this balancing, the therapists choose between continuing silent witnessing on the one hand, and taking steps which will facilitate or resume silent witnessing on the other. These steps are always performed with “empathic presence,” meaning with a non-judgmental, supportive, appreciative, validating, and suggestive manner rather than a directive one (Mithoefer, 2017, p. 9). In virtually all cases, interventions are designed to help the participant stay in touch, or get back in touch, with her unfolding inner experience. Interventions may include comments validating the participant’s reports of her experience including her suffering, suggestions to use “breathing” to “relax into” her inner experience, asking if hand holding or similar nurturing touch may be helpful. If a participant’s anxiety (or fear, shame, anger, guilt, etc.) feels overwhelming, the therapists may encourage her to “stay with” her anxiety to see where it leads, remind the participant that facing difficult feelings is safe in the MDMA-assisted therapy setting and may represent a natural progression of the healing process (Mithoefer, 2017, p. 43).

Throughout, the therapists are asked to employ a “beginner’s mind,” so as not to anticipate what direction the participant’s inner experience may follow, and to preclude efforts to fit the participant’s experience into the therapists’ own theoretical framework.

In addition to suggestions to “use the breath”^3^ to manage challenging thoughts or feelings, therapists may suggest “focused bodywork” should the participant mention a significant somatic experience during an active drug or integration session. Even such a direct relational engagement is discussed in the manual as if the therapist were a resource at the participant’s disposal:

Participant: “Dread and fear were there for so long. You get so used to it, you do not know what it is anymore, especially after having the anxiety disappear. It feels like a whole new wound. It wasn’t the same. It just felt dreadful.”

Therapist: “Would you be willing to explore that or work with that a little bit today? To see what you may discover? Do you feel like you’d like to do some focused bodywork with that lump in your stomach?”

Participant: “Yeah. It is time to try some of that, too.”

Therapist: “It might be a good way to work with it since you know where it is in your body.”

[Later].

At this point, if the participant agrees, they move to either the futon or a mat on the floor with the participant lying down and the therapists sitting on either side.

Therapist: “So maybe just use your breath and breathe into that feeling in your stomach. I encourage you to remain present with whatever comes up. If your body wants to express it in any way, shake, move, or indicate if you want some resistance from us.” (As the focused bodywork was performed, the participant breathed into it and experienced a deep sobbing.) (Mithoefer, 2017, p. 57)

The point I wish to make here is the consistent emphasis in the MAPS inner healing intelligence model on the participant trusting her own resources, including her own body, rather than the intelligence of the therapist, even when direct physical contact with the therapist takes place. The model imputes a sense of agency to the participant precisely when the use of a psychedelic drug contributes to losing one’s sense of agency, a surrender of control. The therapist’s physical being is regarded as another resource to be controlled by the participant’s inner healing intelligence. For individuals with a significant history of trauma, one’s sense of control often lies at the heart of the issue. Dissociation separates oneself from that which it cannot control, yet the need for dissociation implies a lack of control.

The therapists’ emphasis on trusting one’s inner healing intelligence rather than trusting the therapists themselves paradoxically leads to increased trust and openness to advice from others:

I was stressing out, and he was trying to give me advice. And never before could I confidently say that somebody could give me advice, and I would sit there and try to listen to what they are saying. Usually I’m just like, no, you are wrong when you do not understand, but like I was actively believing him and wanting his advice and I was able to see it from that perspective. You know, but like before I was so stuck that I could not listen to what they were saying (Godes et al., 2023, p. 6).

## Is the inner healing intelligence real?

One of the major challenges facing participants and therapists in MDMA-assisted therapy is believing that “inner healing intelligence” is real. Therapists recognize that trauma survivors cannot fully trust its power through pedagogically imparted words. Many participants greet the inner healing intelligence concept with what may be termed healthy skepticism. How can this skepticism be converted into trust?

A prerequisite of trust is consistency. The consistent message from the therapist is that the capacity to heal resides within the participant. In accepting the participant’s skepticism as normal and healthy, the therapist already demonstrates her faith in the process. The therapist’s attitude holds the participant’s skepticism as a necessary part of the autonomous healing process, as an indication, in fact, of the robustness and healing capacity of the participant’s inner healing intelligence. This is a direct and effective solution to the trauma-imposed dilemma of how to ask someone to trust you when their biggest problem is that they simply cannot (Herman, 1992). You say to them, in effect, “We both know you cannot fully trust me even if you wish you could, but I think this treatment process can help you anyway. Each time your doubts arise I will welcome them as an expected part of the process. I will not ask you to stop doubting. I will not try to show you that your doubts are misplaced, prove you wrong, or intrude my own theories into the process. I will only remind you that I do not know exactly what will take place any more than you do (“beginner’s mind”), that the process is different for everyone, that it takes time, and ask without pressuring you if you might be willing to stick with the process a little bit longer.”

The process is not viewed as a magical, drug-induced transformation of doubt but as an outgrowth of the participant’s healthy, self-protective judgment. The same attitude is also implied in the therapist’s approach toward the participant’s fears about losing control. This cuts to the heart of the dissociative process.

Since the early studies of hysteria (Ellenberger, 1970; Breuer and Freud, 1893–1895; Freud, 1893, 1896; Herman, 1992; Perry and Laurence, 1984), the psychodynamic treatment of trauma has traversed the same dissociative terrain that gave rise to symptoms in the first place. This is reflected in the seeming paradox of using dissociative states (hypnosis) or dissociative drugs (ketamine) to resolve dissociative symptoms (Feder et al., 2021). This prospect causes understandable trepidation for the participant, but at the same time the need to confront one’s fears also makes intuitive sense. In effect, the process of psychedelic-assisted therapy rests on the notion of surrendering control in order to gain control. The therapist does not ask the participant to voluntarily surrender control. Because dissociation itself is a process of partially relinquishing control (of one’s customary state of consciousness) in order to stay in control (not to succumb to the annihilation of trauma), the therapist accepts the participant’s ambivalence toward surrendering her usual defenses as further evidence—albeit stunted by the force of trauma—of her inner healing intelligence, even while aligning with the participant’s wish for change by encouraging the participant to “follow the process.”

Thus, the participant’s belief in her own inner healing intelligence evolves from repeated relational transactions in which the therapists’ language and actions affirm their belief in the participant’s capacity to heal herself. The therapists pledge to do no more than be “fully present” with the participant as witnesses and ask no more from the participant than patience and permission to occasionally remind her of her experiences and goals. I suggest, therefore, that *belief in one’s inner healing intelligence is psychodynamically equivalent to trusting the witnessing aspect of the relational process*.^4^ Its reality is supported by the therapists’ steadfast commitment to the participant’s process of healing (Phelps, 2017). In this sense, the participant’s belief in her inner healing intelligence is really just another name—*but the name itself is critical*—for her trust in the therapists’ intention to witness her healing process.^5^ Accessing one’s inner healing intelligence in the MAPS MDMA-assisted therapy protocol enables the trauma survivor to feel witnessed in spite of her shame, reluctance, or fear of being witnessed. Its underpinning in relational trust makes trusting one’s inner healing intelligence *a process of feeling known*, an indispensable step in integrating dissociated aspects of experience.

In addition to working around the participant’s inescapable distrust, this approach takes advantage of the appealing logic of facing rather than avoiding one’s problems while circumventing its chief drawback, having to motivate oneself to experience pain. Instead, one must merely exercise patience and be willing to “follow” a process which is described as emerging spontaneously. Feduccia and Mithoefer (2018) speculate that healing takes place during MDMA assisted therapy through fear extinction via protracted self-directed spontaneous exposure.

As psychedelic subjects are highly attuned to the emotional state of people in their presence (Osmond, 1957; Chwelos et al., 1959; Grof, 1980; Penn et al., 2021) and in a state of heightened receptivity (Carhart-Harris et al., 2018; Dolder et al., 2016; Greenway et al., 2020; Pokorny et al., 2017) or suggestibility (Carhart-Harris et al., 2015), the therapists’ belief in the inner healing intelligence of the participant engenders an eponymous process in the participant. The answer to the question, “Is the inner healing intelligence real?” is that it is as real as the faith the therapists have in the participant’s healing process. This is a big part of the synergy between the subjective effects induced by the drug^6^ (i.e., suggestibility) and the attitude of the therapist. It does not require absolute, unwavering faith from the therapists. If anything, the concept of inner healing intelligence is likely to feel more real to the participant if the therapists acknowledge the very human limits of their faith, but the more openly and candidly these are acknowledged, the more the participant will trust the process, and the more accessible the participant’s dissociated thoughts and feelings will become to feeling witnessed.

## Further relational aspects of the model

Many therapists, including those accustomed to a psychodynamic model, initially may be put off by a model which, on the surface, minimizes the contribution of the therapist to the treatment process. To begin with, there are two therapists, not one. An entire study might be devoted to the multiple ramifications of this fact alone. The stated reasons for this include a safeguard against weariness or fatigue in a process that requires the physical proximity of a therapist for approximately 8 h. It is also a precaution which safeguards a participant who is in no condition to give meaningful consent from even a well-meaning therapist exploiting the participant’s vulnerability under the influence of a powerful drug. In its initial conception (i.e., in the era before notions of gender diversity and fluidity gained prominence), the idea of having one therapist of each gender in the room with the participant was believed to encourage the expression of a wider range of potential transferences, and to afford an option for the participant with a history of sexual trauma to speak directly to a therapist of one gender or the other regarding sensitive or physically intimate memories or feelings.

There are many other ramifications of the two-versus-one therapist format, but as this paper is primarily focused on aspects of the therapist’s attitude in both the MAPS MDMA-assisted therapy and the relational psychoanalytic approach, I will turn directly to the obvious fact that the presence of a second therapist diffuses the dependency field for the participant. This may lessen the pressure an individual therapist feels to be able meet all of the participant’s needs, but it may also diminish the sense of importance the therapist feels about her role in the treatment. This latter effect coincides with the deflationary effect on the therapist’s sense of importance by citing the participant’s inner healing intelligence as the main source of insight and judgment in the process, and by the fact that for extended portions of the active treatment sessions, the participants focus inwardly, wearing headphones to listen to music and eye shades to shut out the world. Most psychodynamic therapists would agree that needing to feel valued by the patient can be a particularly corrosive if common form of countertransference that must be carefully monitored and restrained in an analytic treatment. It is interesting to consider how the MAPS approach influences this form of countertransference. I suggest that to the extent that the therapist buys in, so to speak, to the reality of the participant’s inner healing intelligence, the therapist’s self-esteem is less vulnerable to the destabilizing effects of the participant alternately idealizing and devaluing her as often takes place during challenging treatments, and thus less prone to defensive countertransference enactments.

An additional consideration is how the participant’s taking the psychedelic medicine affects the balance of vulnerability in the room. While the medicine reduces defensiveness in the participant, it also reduces defensiveness in the therapists, as relatively speaking, the participant becomes dramatically more vulnerable than the therapists. This should facilitate the supportive, caring intentions in the therapists relative to their normal protective tendencies in a non-medication-assisted session.

## My bad: Ferenczi and psychoanalytic technique

Ferenczi’s “Confusion of Tongues” paper Ferenczi (1933/1988) marked a tectonic, if gradually recognized, shift in the psychoanalytic theory and technical approach to the treatment of trauma.

The paper is best known for its departure from Freud’s theory of infantile sexuality and the irreparable rift it caused between Freud and Ferenczi (Rachman, 1997; Masson, 1984), but it also described a novel approach to psychoanalytic technique which was ridiculed by Freud. A hint of this technique is embodied in the first four words of Ferenczi’s paper: “It was a mistake…” Ferenczi (1933/1988). Ferenczi goes on to describe how he struggled with many of his patients until he began to listen to many of their spoken and unspoken observations from a new perspective, which might be described as an empathic rather than a strictly analytic point of view. He realized that many aspects of the analytic situation, including its asymmetry with respect to authority, prevented patients from expressing views of their experience in the consulting room “because of a fear of occasioning displeasure in us by their criticism” (p. 198). He considered objectively the idea that in some respects his patients were better analyzed than he was. He recognized the “*professional hypocrisy*” in an analyst’s promising a patient that “we will listen attentively to him, give our undivided interest to his well-being and to the work needed for it” (pp. 198–99), when in fact “we can only with difficulty tolerate certain external or internal features of the patient, or perhaps we feel unpleasantly disturbed in some professional or personal affair by the analytic session” (p. 199). Ferenczi concludes, “I cannot see any other way out than to make the source of the disturbance in us fully conscious and to discuss it with the patient, admitting it perhaps not only as a possibility but as a fact” (p. 199).

Acknowledging the analyst’s contribution to the patient’s inner experience of the analyst broke new ground, both in Ferenczi’s analytic treatments, and in the psychoanalytic understanding and treatment of trauma and dissociation. Previously unanalyzable transference-countertransference enactments could now be symbolized within the analytic dyad.

The traumatic-hysterical attack, even if it recurred, became considerably milder, tragic events of the past could be reproduced in *thoughts* without creating again a loss of mental balance…. Now what brought about this state of affairs? Something had been left unsaid in the relation between physician and patient, something insincere, and its frank discussion freed, so to speak, the tongue-tied patient; the admission of the analyst’s error produced confidence in his patient (p. 199).

What Ferenczi deduced was that the combination of the analyst’s restrained coolness, professional hypocrisy, and covert dislike of the patient created circumstances similar to those under which the patient’s original, often sexual, trauma took place. On top of this strain, placing the responsibility for these circumstances solely on the patient amounted to re-enacting the trauma and reinforcing the patient’s guilt feelings.

By inviting the patient’s expression of critical feelings toward the analyst; by freely admitting one’s mistakes and shortcomings and endeavoring not to repeat them; and by acknowledging the reality of negative countertransferential reactions, the analyst helps the patient build confidence in the analyst. “*It is this confidence that establishes the contrast between the present and the unbearable traumatogenic past*” (emphasis in original; p. 200).

In addition to introducing the technical alterations just mentioned, Ferenczi’s “relaxation therapy” paved the way for contemporary relational psychoanalytic approaches to trauma (Aron, 1992; Mészáros, 2010; Rachman, 2007; Stern, 2021). He favored a flexible, empathically-informed, responsive approach over the inflexible application of analytic abstinence, neutrality and frustration (Rachman, 2007). He sought to project a patient, nurturing, supportive attitude. He encouraged emotional encounters in the analytic situation in which each party’s subjectivity could be openly examined to illuminate the other’s. He favored enactments and expressions of emotion to intellectualized analysis, and recognized that interpretation was as at least as often the cause of submissive compliance and re-traumatization as it was useful for the patient. He was willing to stay present with his patients as long as they needed him, even to a fault (Grubrich-Simitis, 1986; Szecsödy, 2007; Thompson, 1988). In Ferenczi’s words, “The analyst’s behaviour is thus rather like that of a tender mother, who will not go to bed at night until she has talked out with the child all his current troubles, large and small, fears, bad intentions, and scruples of conscience, and has set them at rest” (Ferenczi, 1931, p. 477). All of these modifications served to minimize distrust. Many of these elements of the therapeutic attitude are embodied in the empathic presence approach in MAPS MDMA-assisted therapy. Perhaps it is not surprising then that with his relaxation technique, Ferenczi was able to draw out material from a dissociative state of consciousness that sounds remarkably similar to that which might arise in psychedelic-assisted therapy.

In all free association there is necessarily an element of self-forgetful abstraction; it is true that, when the patient is called upon to go further and deeper in this direction, it sometimes happens—let me frankly confess, with me very frequently—that a more profound abstraction arises. Where this takes a quasi-hallucinatory form, people can call it auto-hypnosis if they like; my patients often call it a trance-state (Ferenczi, 1931, p. 475).

## From Ferenczi to relational theories of witnessing

Over time, there became less and less distinction between Ferenczi’s psychoanalytic technique and his real personality. While for a long time he may have felt an overly-determined need to please his erstwhile analyst and mentor Freud, and may for that reason have been reluctant to acknowledge fully to himself his temperamental differences with Freud (Grubrich-Simitis, 1986; Thompson, 1988), this became less true during the last 6–7 years of his life, when he intrepidly pressed on with his relaxation technique at the risk of losing his mentor’s approval (Rachman, 1997). Thompson notes:

I believe that here at last Ferenczi was seeking to express his own convictions—not Freud’s—for the theory of his relaxation technique was based on his ideas of the child–parent relation. Two beliefs especially influenced his technical attitude—one was the idea that the child needs to feel loved and accepted in order to develop successfully, and the other was that the parent’s attitude toward the child must be sincere (Thompson, 1988, p. 189).

Ferenczi’s relaxation technique not only drew from the failings he experienced with his earlier “active” technique with patients, but deeply reflected his personality and values. It is no coincidence, as I hinted earlier, that his seminal “Confusion of Tongues” paper, which codifies many of the elements of the relaxation technique, begins with the words, “It was a mistake….” In admitting to his patients that he made mistakes with them he was doing no more and no less than he did with an audience of colleagues. His therapeutic technique, while painstakingly elaborated in the crucible of the consulting room and through his interactions with Freud and other colleagues, was in no way contrived. It was centered on the recognition that those who still suffered from trauma needed someone they could trust (Mészáros, 2010). He recognized that beyond technique, it is above all the therapist’s genuineness that inspires trust.

This drove his belief in the importance of acknowledging to his patients the various ways he felt he was failing them. He also recognized that his authentic reactions to his patients in general could be a useful source of information to him and to his patients. This approach, along with his belief in the reality of his patients’ reports of sexual and other abuse, earned Ferenczi’s reputation as the first intersubjective or relational psychoanalyst (Mucci, 2017; Rachman, 2007; Stern, 2021; Szecsödy, 2007).

## Contemporary contributions to the relational model

Benjamin (2009), among others who have built upon Ferenczi’s contributions, engages his approach in her idea of a “moral third.” This principle refers to a space for the two members of an analytic dyad to reciprocally engage in repair processes following inevitable ruptures in their alliance resulting from misattunement. The analyst acknowledges her role in the failure, and in doing so, models a “sense of solidity that can tolerate scrutiny by the other, to transform the complementary see-saw of blame and invite the patient to be an interpreter of the analyst and a co-creator of dialogue, and so develop her own sense of agency and responsibility” (p. 450). The analyst’s acknowledgment of failure is “an action that develops faith in the moral third because it affirms the lawful ethic of responsibility and counteracts past experiences of denial. Such action is meant to show that the analyst can change, can model the transformational process, and that revealing her struggle to do so also transforms the analytic process into one of mutual listening to multiple voices” (p. 450). Here, Benjamin emphasizes that building trust enables witnessing: the analyst’s open acknowledgement of failure *develops faith* in a space (the “moral third”) which transforms the process into one of *mutual listening to multiple voices*. Though the language of MDMA-assisted therapy differs sharply from that of relational psychoanalysis, the efficacy of both processes hinges on the participant/patient’s trust in a named entity (inner healing intelligence or moral third) reached through a treatment process which aims to create space for witnessing.

Poland (2000) describes the analyst as acting as witness when one “recognizes and grasps the emotional import of the patient’s self-exploration in the immediacy of the moment, yet … stays in attendance without intruding supposed wisdom—at least not verbally” (p. 18). He describes the analyst/witness’s listening attitude as marked by “respectful attention on the analyst’s part,” a “silent but active presence,” “engaged nonintrusiveness rather than of abstinence,” and “listening in a way other than to seek for what can be interpreted” (p. 18). He traces the origins of this attitude to empathic responsiveness and the early holding environment, but adds to this “an increasing regard for otherness” (p. 18).

Poland illustrates the nature of analytic witnessing with a remarkable example. A young woman came for her session immediately following what she thought would be a routine gynecologic appointment where she learned that serious pathology had been detected which would require a hysterectomy. She would lose the ability to give birth. Poland characterizes their togetherness as the woman relates her grief. No matter how well-attuned he might be to her grief, the tragic news made clear their “essential otherness” (p. 19). Poland recognized how important it was for the patient to have “someone who understood and *witnessed* her anguish and her efforts to digest her emotional trauma. It was important to her that I see her as a separate real person, one suffering alone a pain that would alter her life” (p. 19).

With a tip to Felman and Laub (1992), Poland acknowledges the truth of Freud’s insight that “it takes two to witness the unconscious.” “For a patient’s testimony to come to life and extend beyond simple conscious access, a comprehending witness is required” (Poland, 2000, p. 20). Analytic witnessing involves recognition, not interpretation, affirmation, admiration, sympathy, or exoneration. The sense of otherness connotes difference rather than sharing, but the implication is that the feeling of otherness itself is intersubjectively shared. From the trauma sufferer’s point of view, the sense of otherness which may accompany witnessing reflects a belief that her experience is grasped frankly, fully. There is no attempt to assuage or soothe, no reframing of her experience or vague offer of hope. The analyst’s “silent but active presence” confirms his primary intention above all to witness; it stems from a recognition that nothing can be done to alter the past, but that witnessing affords a means of preventing isolated horrors from freezing or isolating the past from other self-states (Bromberg, 2000).

Reis (2009) calls attention to the importance of witnessing without ambition, even the ambition to transform dissociated experience into symbolized forms.

I conceive of psychoanalytic witnessing as a living out of traumatic experience in the consulting room, and not as having to do with the expression of warded-off dissociated self-states. Witnessing involves a phenomenon of memory, in what Loewald (1976) termed its enactive rather than representational form. The goal of psychoanalytic witnessing, if there may be said to be a goal, is to allow and witness memory in its varied forms, without attempting to symbolize or make personally understandable the experience – to accept the experience of the experience of trauma, without therapeutic ambition. The analyst occupying the position of witness in a treatment understands that performative and enactive features of traumatic experience are not to be simply translated or transduced into symbolic form, and that a part of the integrity of the experience of trauma is itself its wordless registration (p. 1360).

Reis’s approach evinces an attitude of acceptance and utmost respect for the way in which the analysand conveys the traumatic experience. In Reis’s view, analytic witnessing is an attitude of selfless acceptance that can be conveyed wordlessly.

Davies (1996) conveys a similar respect for the patient’s dissociated traumatic experiences as they are enacted and discussed within the transference-countertransference matrix. “It is not our task to impose meaning on these dyadic systems and emotional schemata, but, rather to stand ready to engage actively with the patient’s internal self and object world and to recognize and confirm meaning when that meaning is offered to us within a shared context” (p. 216).

The relational dynamics which allow dissociated material to emerge through enactment into the transference-countertransference exchange are largely unthought and often result in reactive dissociation in both parties (Bromberg, 1993, 1994, 1996, 2000, 2009; Davies, 1996, 1998; Reis, 2009; Stern, 2003, 2004, 2009a,b, 2022a,b). This can strain the therapeutic alliance. Bromberg (2000) has noted that “for an analysis to be more than a “pseudo-analysis,” the relationship must feel safe, but not ‘perfectly’ safe.” There must be room for “safe surprises” even “collisions” between dissociated self-states of both parties, from which “something new” can arise (p. 15).

In such interpersonal collisions the analyst is often experienced as “going too far,” which, Bromberg tells us, is precisely what enables change to take place. It allows the analyst “the chance to recognize first-hand what “going too far” means, subjectively, to his patient. The relational process through which that recognition takes place is what *negotiating collisions* is all about” (quoted in Greif and Livingston, 2013, p. 338). How this process works depends upon attitude and intention rather than words or cognitions. “What matters is whether patients can feel in an ongoing way his effort to be with them – his effort to keep their dissociated fear and shame in mind while he is *doing* the ‘work’. It is the felt continuity of *being*, especially under adverse conditions, that provides the safety – not a hypothetical capacity to *do* the analysis in some right way” (Bromberg, 2009, p. 358).

Another way the analyst’s attitude influences the relational process is through the perceived intent of his self-disclosure. “The more an analyst’s communication is based on sharing his subjective experience because *he wants it known*, as opposed to wanting it to have a preconceived impact on his patient’s mind, the more it will be felt by the patient as ‘affectively honest’… and the more likely the patient will respond in a similar way” (Bromberg, 2009, p. 358; emphasis in original in both quotes).

While “going too far” and becoming engaged in collisions is a far cry from the MAPS recommended technique for MDMA-assisted therapy, what Bromberg sees as promoting the patient’s sharing (wanting to be known) is the analyst’s affective honesty and his conscientious effort to avoid applying preconceived ideas to the analysand’s experience. The emphasis here on the analyst’s attitude as opposed to *doing* the analysis in some right way is very much in keeping with the themes of empathic presence, genuineness, and “beginner’s mind” that we have seen as critical to facilitating witnessing in the inner healing intelligence model.

## Witnessing and shame

Shame, the feeling related to exposing not-me, is a primary reason for dissociation. Lansky defines one usage of the term shame “as the signal anxiety, arising from conscience that anticipates the imminent danger of rejection, exclusion, unlovability, or disgrace because one will have been found to have failed to conform to standards and ideals. Such a danger is never far away from the trauma sufferer” (Lansky, 2000, p. 140). In this sense, shame may be described as the fear of being witnessed.

A qualitative study found that many patients undergoing MAPS MDMA assisted therapy experienced significant recovery from “moral injury” due to trauma. The authors speculate that the therapy led to an “an increase in acceptance, self-forgiveness, and self-empathy, which are key in addressing moral injury and the feelings of guilt and shame that tie to it” (Godes et al., 2023, p. 8).

In the psychoanalytic situation, the relational process often leads through enactments to the simultaneous experience of shame^7^ in both patient and analyst. In Bromberg’s view, in the seemingly interminable collisions that arise in the enactment of early trauma within the analytic relationship, shame is a sign that “certain of the patient’s dissociated self-states have not been sufficiently acknowledged by the therapist” (Greif and Livingston, 2013, p. 338). These self-states hunger for recognition, but become further cloaked in shame, because the person whose recognition they require cannot offer it as long as he is the person inadvertently causing the shame. These self-states only emerge from dissociation when the analyst comes to a “my bad” moment, and acknowledges his contribution to the collision (Bromberg, 2000; Chefetz and Bromberg, 2004). “If analyst and patient are able to live with it and stay authentically engaged through the many repetitions of the same mess, and the analyst does not try to restabilize himself by invoking the concept of ‘intractable transference resistance,’ something can indeed be done” (Greif and Livingston, 2013, p. 339).

In acknowledging “my bad,” the analyst says, in effect, “I realize now that my trying to view our run-in psychodynamically kept me from acknowledging how frustrated I felt, and kept you from telling me what you really felt.” This is precisely the rationale which informs the therapist’s “beginner’s mind” listening attitude in the inner healing intelligence model. “Empathic listeners are not hesitant to admit they do not have answers” (Mithoefer, 2017, p. 9). The “non-directive approach” emphasizes the need for therapist patience in “allowing participants to come to conclusions themselves” (p. 9).

Before leaving Bromberg’s contribution, I will mention his description of how he writes. He sits down to write, then recognizes that he does not know what to say about his topic. An inner voice urges, “‘So think about it,’” but this only draws a blank. After a taking a break, he returns and still does not know what to say. Then “seemingly unbidden, I start writing.” He stops writing when it begins to feel forced. He later returns, does not read what he wrote, waits until some other unbidden topic comes to mind. After several instances of this, he reads what he has written until he something moves him emotionally. He says to himself, “‘I like this. I wonder what it would be like if I started with this.’ This feels alive.” He then reads through the entire text and finds, or adds with bridging sentences, surprising links between the various bits he has written. “As I keep doing this, little by little, I get a feeling that I know what I’m writing about” (Greif and Livingston, 2013, all quotes p. 336).

As observed earlier regarding Ferenczi (one of Bromberg’s early role models), there seems to be little distinction between the way Bromberg writes and the way he works with his patients. What we can discern in both is his faith in the eventual coherence of the process. In the consultation room, sooner, or later, seemingly unbidden, elements emerge in the relational interchange which enable him to “stand in the spaces” (Bromberg, 1993, 1996) between the patient’s multiple self-states and link them into a cohesive picture. The parallel between Bromberg’s faith in the eventual coherence of the relational interchange and the MDMA-assisted therapist’s faith in the spontaneous unfolding of a process necessary for healing is striking.

## Partners in thought

Stern (2009a) discusses an idea which crystallized as he was watching a film, “The Incredible Shrinking Man.” The protagonist in the film, who is gradually shrinking into nothingness, experiences his life as chaotic, incomprehensible, and meaningless, but his attitude undergoes a remarkable transformation for the better when he decides to narrate his woeful tale into a diary: “I was telling the world about my life,” the shrinking man reads to us from his diary, “and with the telling it became easier” (p. 704). Stern attributes this easing to his having a witness, someone with whom he can share his tragic story. Stern accepts the notion of Poland and others that witnessing is a relational process, but importantly, emphasizes that it may involve an imagined other. The witness may begin as an internalization, “but becomes a changing amalgam of history, fantasy, and current reality” (p. 707). This accords well with the feeling of being witnessed in psychedelic-assisted therapy, where the active but non-directive therapist(s) in the room are just as likely to catalyze a feeling of being witnessed by an “evoked companion” (Stern, 1985) as to serve as witnesses themselves.^8^

For Stern, witnessing is critical in dealing with trauma, but plays a role in normal development as well. He alludes to Fonagy et al.’s mentalization theory (Fonagy et al., 2002), in which the infant’s understanding of her feelings and desires derives from the caregiver’s empathically derived view of the infant, or as Winnicott put it, “What does the baby see when he or she looks at the mother’s face? I am suggesting that, ordinarily, what the baby sees is himself or herself” (Winnicott, 1971a, p. 112). Stern maintains that our earliest self-states are organized around narratives processed through the eyes and ears of one’s caregivers. “We are called into being by acts of recognition by the other” (Stern, 2009a, p. 705).

In Stern’s view, witnessing plays a vital role throughout one’s life. “We need to feel that we exist in the other’s mind and that our existence has a kind of continuity in that mind; and we need to feel that the other in whose mind we exist is emotionally responsive to us, that he or she cares about what we experience and how we feel about it…. Our witness is our partner in thought” (pp. 706–707). The process is so ubiquitous that we notice it mainly when it is disrupted, through misattunement. This is readily observed in the psychoanalytic setting, where the vicissitudes of witnessing are closely scrutinized, and where it is particularly relevant to the process by which “not-me” experience can be transformed to “feels-like-me” experience (Stern, 2003, 2004, 2009a,b, 2022a,b).

This transformation merits closer scrutiny, as it has direct clinical significance. Sullivan coined the term “not-me” to refer to those aspects of experience which are associated with such “awe, horror, loathing, or dread” (Sullivan, 1953, p. 163) that they are sealed off from the rest of one’s identity. Relational psychoanalysts have adopted the term to refer in general to that which is dissociated. Dissociation has been linked to trauma (Kluemper and Dalenberg, 2014; Putnam et al., 1996; van der Kolk and Fisler, 1995), and witnessing has been linked to recovery from trauma (Felman and Laub, 1992; Grand, 2015; Herman, 1998; Richman, 2006).

How does witnessing enable “not-me” to turn into “feels-like-me” in the psychedelic-assisted therapy setting? I have argued that PTSD sufferers in the MAPS MDMA protocol are able to “access” their inner healing intelligence because they accept their therapists’ consistently affirmed commitment to witness their spontaneously unfolding psychedelic experience; that is, when it becomes clear to them that their therapists have no other agenda, no self-esteem need, no theoretical framework system outside of faith in the participant’s own healing capacity supported by the medicine. The movement from “not-me” to “feels-like-me” evolves spontaneously from the participant’s willingness within the relational field to turn inward, which is described as trusting one’s inner healing intelligence.

In Stern’s (2009a, 2012, 2022a,b) account, the process of feeling witnessed requires us to imagine that we exist in the mind of another, which requires that we allow this imagined other to know what we are experiencing. In this way, it is possible to imagine the other’s awareness of ourselves, and it is through this “witnessing” of ourselves that our experiences feel real. During psychoanalytic treatment, our analyst becomes our witness, but not only when we are in our analyst’s presence. Outside the consulting room we continue to “know” our experience by imagining our analyst hearing our stories, which is how we assign them their meaning and come to know how we feel about them.

This account of feeling witnessed may offer a glimpse into the process we call our inner healing intelligence. What I just described occurs as a largely subliminal inner experience. Most of the time that we interact with our partners in thought we are not talking to anybody; we are doing what is called mind-wandering. What guides these seemingly unbidden meanderings? Why not call it our inner healing intelligence. The wisdom of our inner healing intelligence may be construed as *the imagined knowledge our witnesses have, or could have of us if we allowed ourselves to be witnessed*. After all, its “wisdom” cannot be entirely derived from our genetic code alone. It is our partners in thought, or lack of them, which determine what we know and feel, and what we do not know and feel about our experiences, including those which were too horrible to bear. What more trustworthy guide than our way of knowing things?

But as this explanation suggests, our inner healing intelligence is limited by what we allow to be witnessed. According to Stern, dissociation represents “the sequestering of self-states from one another, prevents imaginary witnessing within the personality” (Stern, 2012, p. 61). In a rather far-reaching observation, he states, “The absence of such internal witnessing then prevents the creation of metaphor, because the elements that must combine to make the metaphor—memory and the experience of the present—cannot coexist” (Stern, 2012, p. 61). When trauma causes dissociation, “internal witnessing” does not take place. This freezes memory from contextualization with subsequent experience, prevents metaphor, *constrains free association*, and therefore forecloses the “creative use of traumatic experience” (Stern, 2012, p. 61; see also Stern, 2017; Richman, 2013).

## Psychedelics, dissociation, and self-with-other relations

How do psychedelic drugs influence access to dissociated aspects of experience? Letheby and Gerrans’s (2017) “unbinding” model may shed light here. The model makes use of binding (Sui and Humphreys, 2015) and predictive processing theories (Friston, 2010). According to these theories, a hierarchically structured brain generates “top down” probabilistic inferences about the likely causes of “bottom up” sensory information. Features which regularly associate over time are bound together as coherent percepts. Their recurring, unified features are inferred to be properties of objects. The self is postulated as one of these objects “in order to make sense of, unify, and predict ongoing patterns of egocentric, salient, autobiographical experience” (Letheby and Gerrans, 2017, p. 4). Letheby and Gerrans postulate that the phenomenology of psychedelic-induced ego dissolution, in which the seat of consciousness is no longer bound to various aspects of the self-model, including ownership of body parts, emotions, and autobiographical continuity, follows from psychedelics disrupting the brain’s binding mechanisms. With “significance” no longer guided by what matters to one’s self, the brain is freed from egocentric considerations in attributing salience to sensory and imagined phenomena. New meanings untainted by the self-narrative are apprehended (Hartogsohn, 2018).

Translating this into an object relations framework, in which the relevant predictive models are not isolated constructs of self *or* objects (others) but models of *self-with-other* (Stern, 1985; Davies, 1998), what psychedelics do is disrupt the binding mechanisms of predictive self-with-other models, models which during ordinary states of consciousness form the basis for anticipating outcomes of real or imagined interactions. This may be the basis for the frequently observed decrease in, or even complete loss of defensiveness in psychedelic states (Eisner and Cohen, 1958; Fischman, 2019: Grinspoon and Bakalar, 1986), and the marked increase in suggestibility (Carhart-Harris et al., 2015). Disrupting the brain’s binding mechanisms frees present self-with-other interactions from constraints of prior expectations. In other words, when we see ourselves through the eyes of others, what we imagine the other sees is no longer prejudiced by prior ideas about the other’s perspective. This opens a door *to feeling witnessed*.

The term “dissociation” is used in many different ways in the psychological and psychoanalytic literature (Schimmenti and Caretti, 2016). Putnam (1997, p. 152) uses dissociation to refer to the non-integration of discrete behavioral states resulting from traumatic disruption. Psychodynamically, it has been construed as a defense, implying it has an adaptive function which protects the self, or more accurately, the rest of the self from overwhelming stimuli or psychic pain (Young, 1988). Mitchell (1991) and Bromberg (1996), among other relational psychoanalysts, view the unity of the self as an adaptative illusion which obscures an earlier developmental stage of a multiplicity of selves or self-states. Dissociation is a fallback position in the face of threat. “When the illusion of unity is too dangerous to be maintained there is then a return to the simplicity of dissociation as a proactive, defensive response to the potential repetition of trauma” (Bromberg, 1996, p. 4^9^). One effectively sequesters such experiences and their unbearable associated affect from the self-concept, renders them as “not-me.”

These dynamics suggest another aspect of the synergy between medication and therapy in the psychedelic-assisted treatment of trauma. Increasing integration between self-states is a generally accepted treatment goal in trauma therapy. Unformulated or dissociated self-states enter into psychoanalytic treatment mainly in the form of enactments. Such enactments often threaten the therapeutic alliance by inducing overwhelming affect in both parties, but also afford a critical opportunity for integration. The trouble is that both analysand and analyst get self-protectively stuck in behavioral trenches shaped by their expectations and are thus enjoined from seeing and feeling each other in new ways. Interpersonal witnessing is precluded, especially in configurations of simultaneously occurring dissociated shame discussed above. Psychedelics, by unbinding abstract models of self-with-other relations, prevent the participant from generating expectations modeled upon prior experience, including expectations that the analyst’s response will reinforce her sense of shame. Absent entrenched expectations, new self-with-other relations are forged.

## Psychedelic therapy as a waking dream

While psychedelics may prevent the kind of interpersonal collisions discussed above, it is still unclear how psychedelics facilitate the emergence of dissociated experience within the therapeutic dyad. Here, the relevant model is the dream. How one thinks and feels in the psychedelic state is guided by many of the same principles that obtain while one is dreaming. The subject of experience, or the seat of consciousness in a psychedelic state, like that of the dreamer, is no longer identified with the narrative self, which to some degree no longer exists. Metaphorically speaking—but under the primary process the metaphoric *as if* relationship condenses as word presentations regress to thing presentations—one stands outside one’s narrative self, observes one’s self from a third party perspective (Fischman, 2022). In dreams, the suspension of the secondary process allows one’s self-representation to be a character among other characters or object representations in a narrative. As in a dream, the psychedelic subject of experience links “day residues” (Freud, 1900) or recent engagements with the world to the real or imagined experiences that their physical qualities (“thing presentations”) or emotional valences bear resemblance to.

In the psychedelic-assisted therapy situation, the relevant day residues are precisely the set and setting of the drug experience. This includes goals and intentions discussed by the participant and therapist in preparation for the drug session, such as working through trauma. The psychedelic experience, like the dream, becomes a virtual reality generator for processing the elements of set and setting in new configurations simulating social or threatening situations (Revonsuo, 2000; Revonsuo et al., 2015).

Here, the synergy between drug effect and psychotherapy is again apparent. The chief difference between the dream and the psychedelic-assisted therapy situation is that one stays awake throughout the latter. Whereas sleep largely cuts off the dreamer’s exposure to her setting, the psychedelic subject is continuously exposed to the influence of setting. Writers have noted the extreme sensitivity of the psychedelic subject to sensory input or setting (Carhart-Harris et al., 2018; Jobst et al., 2021), which takes on a quality of immediacy like that which occurs at the onset of psychosis (Klee, 1963; Salzinger et al., 1970). In this state of heightened sensitivity, the psychedelic subject of consciousness receives ongoing input from her setting, namely therapists who embody the subject’s intentions to work through trauma-induced feelings. Mithoefer notes, “In MDMA-assisted psychotherapy we have an agreement with participants that the therapists can bring up the index trauma at some point during each MDMA-assisted session if it does not come up spontaneously, but in almost 100 MDMA research sessions to date, we have never had to do so” (Mithoefer, 2017, p. 70). This “spontaneous” emergence of trauma-related content likely reflects the continuous influence of setting (therapists’ attitudes and intentions) in a medication-induced state of heightened sensitivity. Under such conditions, the previously “frozen” process of memory consolidation (Stern, 2012), *now guided by primary process and metaphor in a waking state*, makes traumatic experience available for creative use in the treatment setting (Stern, 2022a).

## The conveyor belt

Laub (1991) has recorded a remarkable example of the way a setting dedicated to witnessing can serve as a day residue in a dream, which can creatively re-contextualize previously frozen traumatic experience. Laub presents the case of high-ranking Israeli military officer whom he invited to give testimony at the Video Archive for Holocaust Testimonies at Yale. As a 5-year-old child in the Krakow ghetto, he overheard his parents at night discussing a plan to smuggle him out of the ghetto in advance of an anticipated roundup of children for extermination. One night they wrapped him in a shawl and smuggled him outside the gate, gave him a passport photograph of his mother as a student. His mother advised him turn to the photograph whenever he needed to, and promised that his parents would find him when the war was over.

He stayed at a sex-worker house whose address his parents had given him, which seemed to him like a hospital. Later he stayed with a gentile family where the matriarch allowed him to pray to his photograph of his mother while the others knelt before a crucifix. He prayed that his mother would take him back after the war was over, as she had promised. He never doubted that this would happen.

Laub refers to the photograph as his first witness.

At the end of the war, his parents did indeed come back to him, but they were not the parents he kept alive in his mind with the help of the photograph. They were haggard, emaciated death camp survivors in striped uniforms. He was devastated and began having regular nightmares of being on a conveyor belt moving relentlessly toward a metal compactor that will crush him. He awakened from these nightmares in a terror, disoriented. The sustaining effect of the photograph, his witness, was erased by the new image of his victimized parents, whom he could not bring himself to call Mom and Dad.

Laub describes this phase of his life as typifying that of a child victim deprived of the “holding” presence of a witness. He went on to become a soldier known for fearlessness, and acted on the battlefield as if he were invulnerable. Laub views this as a defense against seeing himself as a victim.

Laub’s invitation to give testimony provoked a crisis. He was very reluctant to accept, but his wife pointed out to him that years of avoiding talking about his past had not diminished his regular nightmares. He resolved to give testimony. Following this decision, he had the nightmare again, but for the first time, he stopped the conveyor belt. He woke up anxious, but this quickly gave way to feelings of fulfillment and satisfaction. He was not disoriented. “I knew where I was. I knew what happened” (Laub, 1991, p. 89).

## The therapist’s attitude and witnessing: conclusion

The story of the soldier also illustrates the different ways we can acquire and lose our witnesses. Stern’s “partners in thought” can be actual people, internalized images of people, imagined interested parties, readers of a memoir (Richman, 2006), some amalgam of the above and more. Witnessing takes place in a numinous space located between inner and outer, between subjectivity and objectivity, what Winnicott (1971b) called transitional space. It is here that the transition from “not me” to “feels-like-me” transpires.

In its essence, a witness is an interested other who is committed to knowing someone’s story. When a subject is assured of this, testimony will follow, because it is only through another knowing our story that we can be sure it happened. Trauma is often unavoidable, but post-traumatic dissociation may be mitigated by witnessing. The absence of a witness feels like death (Boulanger, 2012; Gerson, 2009). The trauma survivor is caught between wanting to know and be known on the one hand, and shame and mistrust on the other. The model of the inner healing intelligence is simple and appealing to someone caught in this bind. It is a model of being one’s own partner in thought. Telling a story to oneself is far less imposing than finding a witness.

As I hope to have made clear, the inner healing intelligence is a process of witnessing. The task for the therapist in both psychedelic-assisted therapy and in relational psychoanalytic therapy is to ratify the subject’s mistrust, shame, suffering, even spiritual “death;” to accept and acknowledge one’s limitations and failings in attempting to witness; to remain interested and patient for as long as necessary to allow the subject to create or discover a partner in thought through whom she can know herself.

To conclude this discussion on a lighter note, the attitude that the MAPS MDMA therapist and the relational psychoanalyst have in common is: “My bad, you got this!”

## Data Availability

The original contributions presented in the study are included in the article/supplementary material, further inquiries can be directed to the corresponding author.

## Footnotes

^1^Since this paper was originally submitted, the FDA has requested an additional Phase 3 trial instead of approving the Lykos (MAPS) application for approval of MDMA-assisted therapy for PTSD. While reasons for the decision have not been made public, a significant limitation of the studies has been the difficulty in insuring true blindness for both participants and therapists due to the prominent subjective effects of the drug. The study authors acknowledge the possibility of expectation bias affecting positive outcomes.

^2^This paper adds that the MAPS MDMA-assisted therapy approach also recognizes the importance of the relational context in which the participant’s inner healing intelligence acts, which is exactly what will be discussed here, though with a different emphasis.

^3^The manual notes there is an art to the way language is used. The gerund “breathing” is preferred to “breathe” because it is more suggestive than directive (p. 10). In the same way, I suspect there is art to the manual’s use of the term “the breath” when it is to be employed as a means of engaging feelings, as it makes the act of breathing sound like an additional personal resource one can deploy to manage challenging feelings.

^4^The MAPS MDMA-assisted therapy team leaders have come close to acknowledging this. “Although it is frightening for many of us as helpers to witness, allowing the inner healer to resurrect the trauma and allow it to express itself and ultimately be integrated is just another part of trusting the process” (Razvi, S. in Mithoefer et al., 2014), but continue to refer to inner healing intelligence as a psychic agency.

^5^Trusting one’s inner healing intelligence bears resemblance to Winnicott’s (1958) capacity to be alone, which paradoxically, takes place in the presence of the mother, whose orientation has been devoted to the infant’s ego requirements. One may say that a parallel orientation of the therapist in the MDMA-assisted therapy setting enables the participant to be alone with her inner healing intelligence, paradoxically in the presence of another.

^6^MDMA enhances trust (Grinspoon and Bakalar, 1986), perhaps by unbinding protective self-with-other models (see below).

^7^Shame is powerfully associated with trauma related distress (DeCou et al., 2023; Lansky, 2000; van der Kolk et al., 2023), and particularly with interpersonally violent trauma (Budden, 2009).

^8^These two forms of feeling witnessed in psychedelic-assisted or conventional psychotherapy are not mutually exclusive. The witnessing attitude in the therapist undoubtedly promotes access to imagined self-with-other interactions with evoked companions in the participant.

^9^Page number in electronic version: https://archive.wawhite.org/uploads/PDF/E1f_5%20Bromberg_P_Standing_in_the_Spaces.pdf.
